# Methodological and Statistical Considerations for Cross-Sectional, Case–Control, and Cohort Studies

**DOI:** 10.3390/jcm13144005

**Published:** 2024-07-09

**Authors:** Edsaúl Emilio Pérez-Guerrero, Miryam Rosario Guillén-Medina, Fabiola Márquez-Sandoval, José María Vera-Cruz, Martha Patricia Gallegos-Arreola, Manuel Alejandro Rico-Méndez, José Alonso Aguilar-Velázquez, Itzae Adonai Gutiérrez-Hurtado

**Affiliations:** 1 Departamento de Biología Molecular y Genómica, Instituto de Investigación en Ciencias Biomédicas, Centro Universitario de Ciencias de la Salud, Universidad de Guadalajara, Guadalajara 44340, Mexico; 2 Doctorado en Farmacología, Instituto de Investigación en Ciencias Biomédicas, Centro Universitario de Ciencias de la Salud, Universidad de Guadalajara, Guadalajara 44340, Mexico; 3 Doctorado en Ciencias de la Nutrición Traslacional, Departamento de Clínicas de la Reproducción Humana, Crecimiento y Desarrollo Infantil, Centro Universitario de Ciencias de la Salud, Universidad de Guadalajara, Guadalajara 44340, Mexico; 4 Departamento de Biología Molecular y Genómica, Instituto de Nutrigenética y Nutrigenómica Traslacional, Centro Universitario de Ciencias de la Salud, Universidad de Guadalajara, Guadalajara 44340, Mexico; 5 División de Genética, Centro de Investigación Biomédica de Occidente (CIBO), Centro Médico Nacional de Occidente (CMNO), Instituto Mexicano del Seguro Social (IMSS), Guadalajara 44340, Mexico; 6 Doctorado en Genética Humana, Centro Universitario de Ciencias de la Salud, Universidad de Guadalajara, Guadalajara 44340, Mexico; 7 Laboratorio de Ciencias Morfológico Forenses y Medicina Molecular, Departamento de Morfología, Centro Universitario de Ciencias de la Salud, Universidad de Guadalajara, Guadalajara 44340, Mexico; 8 Departamento de Biología Molecular y Genómica, Centro Universitario de Ciencias de la Salud, Universidad de Guadalajara, Guadalajara 44340, Mexico

**Keywords:** cross-sectional studies, case–control studies, cohort studies, methodology, statistics

## Abstract

Epidemiological studies are essential in medicine and public health as they help identify risk factors and causes of diseases. Additionally, they are key to planning, implementing, and evaluating health interventions aimed at preventing and controlling the spread of diseases. Among these studies, analytical observational studies, such as cross-sectional, case–control, and cohort studies, are the most used. The validity of their results largely depends on the robustness of the design, execution, and statistical analysis. **Objective:** The objective of this study is to examine the most common errors in the selection of methodological design and statistical tests in analytical observational studies and to provide recommendations to correct them. **Methodology:** A comprehensive review of the available literature on methodology in epidemiological observational studies was conducted, focusing on cross-sectional, case–control, and cohort studies. Common errors in the selection of designs and statistical tests were identified and analyzed. **Results and Conclusions:** Errors in the selection of methodological design and statistical tests are common in epidemiological observational studies. Based on the identified errors, a series of recommendations is provided to improve the selection of methodological design and statistical tests, thereby increasing the reliability of the results in cross-sectional, case–control, and cohort studies.

## 1. Introduction

Epidemiology is a discipline that focuses on the study of the occurrence, distribution, and determinants of diseases in human populations. It relies on epidemiological studies, which, through investigations guided by the scientific method, allow us to understand the distribution of diseases, identify their causes and risk factors, and develop methods for their control and prevention [1,2]. Epidemiological studies are classified as descriptive and analytical. Descriptive studies record the occurrence of diseases and enable the determination of prevalence, incidence, and temporal patterns without making comparisons between groups to infer causality or associations of risk factors with health conditions [3]. On the other hand, analytical studies seek to estimate the relationship between variables, i.e., how an exposure (considered as an independent variable) relates to an outcome (considered as a dependent variable). Unlike descriptive studies, analytical ones focus on making comparisons between groups to infer causality or associations between risk factors and health conditions [4,5]. This paper focuses on the analysis of key points to develop and evaluate the three most common types of analytical observational studies: cross-sectional, case–control, and cohort studies [2].

While there are currently articles that serve as guides to increase the quality of analytical observational studies and guidelines designed to improve transparency in the reporting of epidemiological studies, such as the STROBE Statement (Strengthening the Reporting of Observational Studies in Epidemiology), many publications still present significant opportunities for improvement necessary to ensure the quality of the results of epidemiological studies [2,6,7,8]. In this review, the most common errors in the selection of case–control studies, cohort studies, and cross-sectional studies, as well as in the choice of appropriate tests for statistical analysis, are identified and analyzed. Based on this analysis, practical recommendations are offered aimed at improving the accuracy in the selection of statistical analyses, the validity of the results, and facilitating the appropriate choice of study design.

## 2. Methods

The objective of this review was to identify the most common errors in the selection of statistical tests and in the classification of case–control studies, cohort studies, and cross-sectional studies to provide recommendations to improve accuracy and validity in the categorization of these epidemiological research designs.

To achieve this objective, a literature search was conducted in the PubMed and Web of Science databases between December 2023 and February 2024. The following keywords were used: “common errors” AND “classification” AND “case-control studies”, “common errors” AND “classification” AND “cohort studies”, “common errors” AND “classification” AND “cross-sectional studies”, “methodological errors” AND “case-control studies”, “methodological errors” AND “cohort studies”, “methodological errors” AND “cross-sectional studies”, “classification issues” AND “epidemiological studies”, “errors in study design” AND (“case-control” OR “cohort” OR “cross-sectional”), “misclassification” AND “epidemiological studies”, “bias” AND “errors” AND (“case-control” OR “cohort” OR “cross-sectional”), and “challenges” AND “classification” AND “epidemiological studies”.

The selection of articles was conducted through a comprehensive review. Articles in English and Spanish that addressed errors in the selection of methodological designs and statistical analyses in case–control, cross-sectional, or cohort studies, as well as those offering solutions to the identified problems and useful for the development of such studies, were included.

The article selection process comprised several stages. First, an initial screening was conducted by reviewing titles and abstracts to assess the relevance of the retrieved articles. Subsequently, a full review of the selected articles was conducted, in which their relevance to the objective of identifying and analyzing common errors in epidemiological studies and their statistical analysis was evaluated. Then, relevant data on the identified errors were extracted, along with information aimed at improving accuracy and validity in the categorization and statistical analysis of the studies. Finally, the extracted data were synthesized to provide an overview of the most common errors, and based on this synthesis, a set of practical recommendations was developed to facilitate the correct choice of methodological design and relevant statistical tests for an epidemiological study.

## 3. The Proper Classification of Analytical Observational Studies

The value of research findings is linked to the strengths and weaknesses in the design, execution, and analysis of the study. Errors in the classification of analytical observational studies are common and can have a significant impact on the interpretation and application of findings. Inadequate classification of a study can lead to inappropriate methodologies, miscommunication of results, and incorrect conclusions about the study’s effects. These issues not only affect the validity of research but also have important implications for evidence-based medical practice and public health [2,6]. Additionally, article retractions are often associated with problems in statistical analysis or the selection of methods [9,10,11].

Currently, multiple publications have identified that cross-sectional, case–control, and cohort studies are misclassified. To provide an overview of the frequency of misclassification, the results of the misclassification analysis of observational analytical studies are presented in Table 1.

In addition to classification errors, some publications categorize research by mixing designs with different methodologies, for example: “prospective cross-sectional case-control study”, “case-control cohort study”, “retrospective matched cohort case-control study”, “repeated measures case-control study”, or “prospective double-blind randomized case-control study” [13].

## 4. Key Points for Correctly Classifying a Study

### 4.1. In Cross-Sectional Studies, Exposure and Outcome Are Identified Simultaneously

A cross-sectional study is a type of observational study that can be classified as analytical or descriptive. Descriptive cross-sectional studies focus on determining the prevalence of one or more health conditions in a specific population. On the other hand, analytical cross-sectional studies seek to simultaneously evaluate the relationship between an independent variable (exposure) and a dependent variable (outcome) in a specific population. The main characteristic of an analytical cross-sectional study is that it collects data on both exposures (risk factors or independent variables) and outcomes (such as disease or dependent variables) at a single point in time. These studies are traditionally described as a ‘snapshot’ of a group of individuals [5,17]. Since the independent and dependent variables are measured simultaneously, a cross-sectional study cannot establish causal relationships but rather provide a measure of association [17,18].

Unlike other research designs, such as case–control studies, in cross-sectional studies, the study population is not selected based on the dependent variable or outcomes. In cross-sectional studies, participants are chosen according to the inclusion and exclusion criteria established in the study without considering exposure (independent variable) or outcome status (dependent variable) in the selection of the study population. Once the study population is assembled, exposure and outcomes are evaluated simultaneously and classified for analysis [12,19]. In cross-sectional studies, when prevalent cases are included, the measure of an association called odds ratio (OR) becomes the prevalence odds ratio (POR), and instead of the risk ratio (RR), the prevalence ratio (PR) is calculated. Although the mathematical calculations are the same for OR and RR, the interpretation differs in cross-sectional studies [17,20].

To contextualize the key points of a cross-sectional study, one can consider an article published by Małgorzata Biernikiewicz and colleagues. This study aimed to investigate whether there is an association between obesity and erectile dysfunction in men diagnosed with coronary artery disease. To this end, a specific population for the study was selected, consisting of men aged 60 years or older diagnosed with coronary artery disease. Once this population was established, the variables of interest were evaluated in a single measurement. Obesity (independent variable) was defined using body mass index (BMI) and waist circumference as measures, while erectile dysfunction (dependent variable) was assessed using the International Index of Erectile Function 5 (IIEF-5). For analysis, participants were grouped according to the results of the variable measurements, and finally, these groups were compared to establish the association [21].

### 4.2. In Cross-Sectional Studies, There Is Neither Prospective nor Retrospective Follow-Up

In cross-sectional studies, there is no longitudinal tracking; hence, there is neither prospective nor retrospective follow-up [17]. In cross-sectional studies, the measurement of the unit of analysis is conducted at a single point in time. In contrast, in longitudinal studies, whether retrospective or prospective, there is continuous monitoring of a group of individuals over time, often involving the measurement of variables of interest on multiple occasions. This allows for examining how variables change or develop over time in the same sample of individuals [22,23].

Given the above, it is not appropriate to designate a cross-sectional study as retrospective or prospective, as this would be equivalent to stating that a study is both cross-sectional and longitudinal. It is crucial to highlight this point, as the number of publications classified as “prospective cross-sectional” or “retrospective cross-sectional” studies has been increasing in recent years, as reflected in Table 2.

There is a type of cross-sectional design known as “repeated cross-sectional,” which could be confused with a longitudinal study and is sometimes referred to as “pseudolongitudinal”. In this type of study, researchers collect data from a target population at different points in time. The main distinction from a cohort study lies in that, in a cohort study, the same individuals are continuously followed over time, whereas in a repeated cross-sectional study, different samples of individuals are taken on each occasion. In this way, repeated cross-sectional studies allow for the analysis of changes in the population over time [17,25,26].

Repeated cross-sectional studies are very useful for analyzing population changes over time. For example, Anita Siller and colleagues used a repeated cross-sectional study to evaluate the seroprevalence of antibodies against SARS-CoV-2 in the state of Tyrol, Austria. Their findings indicated an increase in mean antibody levels during the SARS-CoV-2 waves of winter 2021/2022 in the studied population. In this type of study, continuous follow-up of the same individuals is not conducted; instead, during each SARS-CoV-2 wave, data from different people within the same target population can be included [27].

### 4.3. In Case–Control Studies, the Study Population Is Selected Based on the Outcomes

Case–control studies are based on the presence or absence of a disease, outcome, or event of interest. In these studies, individuals who have the outcome (cases) are compared with those who do not (controls). Typically, these studies are retrospective, as researchers analyze which individuals were exposed to a specific risk factor in the past. These studies are particularly appropriate when two criteria are met: First, they are appropriate when the outcome or disease studied is uncommon. This is because, in rare diseases, it would be necessary to study many people to find enough cases. A case–control study is more efficient because it starts with already identified cases and seeks an adequate number of controls, reducing the necessary sample size, cutting costs, and allowing the research to conclude in less time. Second, they are appropriate when there is reliable evidence of past exposure [28,29,30].

An appropriate example of a case–control study is the one conducted by Naiara Martínez-Pérez and colleagues. The study aimed to investigate the association between red meat consumption and variants of the *N*-acetyltransferase 2 (NAT2) gene with colorectal cancer. To do this, they carried out a case–control study that included 229 patients diagnosed with colorectal cancer and 229 age- and sex-matched controls. The selection of groups was based on the outcome event or dependent variable, in this case, colorectal cancer. Subsequently, in both groups, independent variables were identified, namely, genotypes and dietary patterns [31].

This type of design is particularly suitable in this case, as it compares two groups based on the event of interest, which in this case is colorectal cancer, seeking to retrospectively identify associated risk factors. Although there are prospective cohort studies in the literature that analyze the association between red meat consumption and colorectal cancer, these studies typically involve a considerably large population (thousands of people) and require follow-up over several years [32,33,34].

Notably, some publications that self-identify as case–control studies are clinical trials. In a review of 28 articles labeled as case–control studies, it was found that approximately 20% were actually intervention studies [35]. Case–control studies are observational designs where there is no manipulation of variables by the researcher; any study that manipulates the independent variable cannot be classified as a case–control study [36,37].

### 4.4. Cohort Studies Compare Study Groups Based on the Risk Factor

In cohort studies, participants are selected based on exposure to a specific risk factor (independent variable), and they are followed up to observe the development of an outcome (dependent variable). With cohort studies, it is possible to examine multiple outcomes based on exposure to a certain risk factor. Cohort studies are preferred for determining the incidence and natural history of a disease and have the potential to provide stronger scientific evidence than case–control and cross-sectional studies [38,39].

Cohort studies are necessarily longitudinal and can be classified as prospective or retrospective. A simple way to identify whether it is prospective, or retrospective is through the outcome of interest. If the outcome has not occurred at the start of the study, then it is a prospective study; however, if the outcome is present at the beginning of the research, then it is a retrospective study [38].

A good example of a cohort study is an article published by Maddalena Alessandra Wu and colleagues, whose objective was to evaluate complications of venous thromboembolism secondary to COVID-19. To do this, they selected two groups based on the risk factor: one group consisting of patients with venous thromboembolism associated with COVID-19 and another group with venous thromboembolism not related to COVID-19. Subsequently, a follow-up of several months was conducted, and dependent variables or outcomes, such as response to drugs and complications, including chronic thromboembolic pulmonary hypertension, bleeding events, and recurrence of the disease, were evaluated and compared between the groups [40].

### 4.5. How to Differentiate between a Retrospective Cohort and a Case–Control Study?

It has been reported that case–control studies and retrospective cohort studies are relatively frequently confused [13,16]. This confusion likely stems from the similarities between the two types of studies, as both investigate data from past events as well as the relationship between exposures and outcomes. In the case of case–control studies, previous exposure is compared between cases and controls, whereas in retrospective cohort studies, outcomes are compared between groups defined as exposed and unexposed. The key point in identifying a retrospective cohort study is that exposure or lack of exposure defines the inclusion criteria, and different outcomes can be obtained based on a specific exposure. Conversely, in case–control studies, the inclusion criteria are defined by the dependent variable or the outcome of interest, and from there, different possible risk factors can be identified [16].

To better contextualize the differences between a retrospective cohort study and a case–control study, it is useful to examine some recent publications. In 2024, Elena Mellado-García and colleagues published a retrospective cohort study aimed at “evaluating and comparing the effects of using a bathtub vs. a therapeutic shower during labor on pain perception, the use of epidural analgesia, labor duration, and maternal and fetal outcomes”. In this study, the exposure (therapeutic shower) was known for all participants from the beginning, and various outcomes (pain perception, use of epidural analgesia, labor duration, and maternal and fetal outcomes) were subsequently investigated [41].

In case–control studies, the comparison is made based on the dependent variable or outcome, and the distribution of exposure is not known from the beginning. An example of this type of study was published in 2024 by Vasiliki Rengina Tsinopoulou and colleagues. Through a case–control study, they investigated factors associated with early menarche in Greek girls. They compared two groups, girls with early menarche vs. girls with normal menarche, and retrospectively looked for risk factors [42].

As can be seen in the examples, in retrospective cohort studies, the analysis starts with the exposure or risk factor, and from this, multiple outcomes can be evaluated. In contrast, a case–control study begins with the outcome or event of interest and retrospectively identifies possible risk factors.

## 5. Statistical Considerations in Analytical Observational Studies

Statistical analysis is an essential element in all research. The proper selection of statistical tests ensures the validity and reliability of the obtained results. However, aligning these tests with the scope, type of study, and variables under consideration can be a real challenge [43,44,45].

The use of inappropriate statistical tests is a common problem in health research [46]. Regardless of the specific area, the frequency of errors in selecting appropriate statistical tests has been identified as high. In a review published in 2005, statistical errors were found in 71% of 83 publications in the field of urology (mainly cross-sectional and cohort studies), with the most common error being the use of incorrect tests for the type of data [47]. Another review published in 2019 analyzed 83 case–control studies in the field of orthopedics, finding that 51% of them used inappropriate statistical methods in their analyses [48]. For this reason, this section presents strategies for the effective selection of statistical tests, considering the study design and the nature of the variables involved.

**Figure 1:**
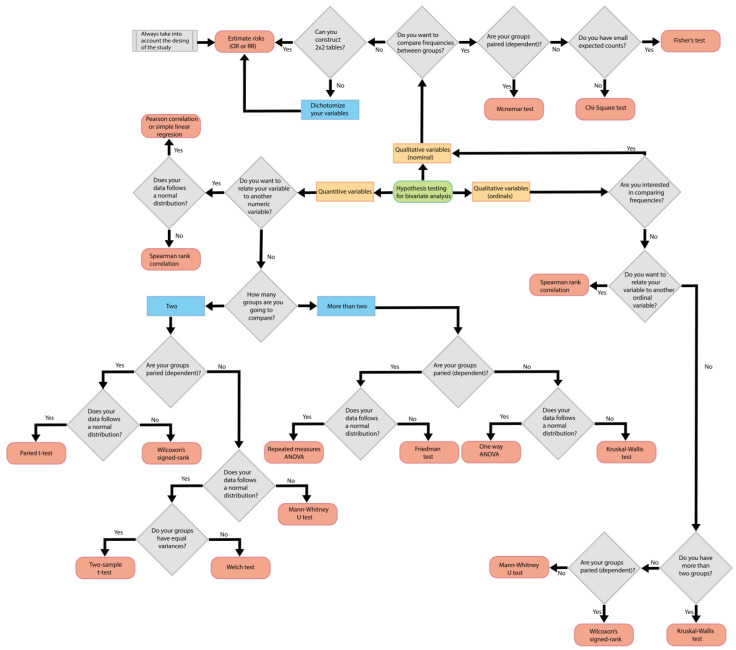
Decision flow for selecting the most used statistical tests in cross-sectional, case–control, and cohort studies. This diagram is designed to assist researchers and reviewers in selecting and applying a test based on the variable type, the relationship sought with the variable, the nature, and the number of groups. The rationale for using this diagram is to start at the center of the figure at “Hypothesis testing for bivariate analysis” and then select the type of variable and continue with the flow of questions until a test is selected. The design proposed for this figure is that the answers are mutually exclusive and only one result can be selected. In all these decisions, it is essential to ensure that the data meet the assumptions of the selected tests. This includes normality of distribution, homogeneity of variances, and sample size, among others. It is also important to remember that this decision flow is a general guide and that there may be specific situations where more detailed consideration or consultation with experts is required. Remember that there may be exceptions and that this diagram does not consider the type of statistical test for each study but generalizes the most used tests in the epidemiological designs under review.

To identify the most appropriate statistical tests in a research study, it is crucial to begin by correctly identifying the types of variables, understanding the dynamics of the groups, and precisely defining the research objectives. To this end, we propose the following six simple steps, which are complemented by a diagram illustrated in Figure 1 (located at the end of point 5). These steps include the following:

1. Identify the type of variables.
2. Determine if the statistical test involves comparing between groups or intends to find the relationship with another variable.
3. Determine if measurements will be taken in different groups or if repeated measurements will be taken in the same group over time.
4. Establish the number of groups being compared.
5. Identify the distribution of the data.
6. Define the type of study.

Starting from these points, it is easy to identify the most appropriate statistical tests for a research study. It is crucial to note that this work focuses solely on hypothesis testing used in frequentist statistics. It includes the most used tests in the literature for cross-sectional, case–control, and cohort studies without delving into aspects such as regressions, multivariate and multivariable analysis tests, or aspects related to Bayesian statistics. Below is a more detailed description of these essential points for selecting a statistical test.

### 5.1. Identify the Type of Variables

The first step involves identifying the type of variable to compare. At this point, it is necessary to determine whether the research studies a numerical variable (either continuous or discrete) or a qualitative variable (nominal or ordinal) [49].

Qualitative variables are divided into two main categories: nominal and ordinal. Nominal variables represent characteristics that do not have an inherent order and, therefore, cannot be classified in a specific order. For example, marital status is a nominal variable, as the categories (single, married, divorced, etc.) do not have a predetermined order. On the other hand, ordinal variables have a natural order, but the differences between categories may not necessarily be equal. For example, a pain measurement scale could be used to determine the categories of no pain, mild pain, and severe pain. Although these categories have an order (from least to most pain), the differences between them may not be uniform [49].

In the analysis of qualitative variables, the most common statistical tests include the Chi-Square Test, Fisher’s Exact Test, and McNemar’s Test [50,51,52]. In cases where the variable is ordinal, such as rating scales, tests such as the Mann–Whitney U test, Kruskal–Wallis test, and Wilcoxon’s signed-rank test can be used [53]. Finally, when the objective is to investigate the relationship between two qualitative variables, our options are limited to certain risks and specific statistical considerations [54].

On the other hand, when analyzing quantitative or numerical variables, the options are broader. Tests such as the two-sample *t*-test, paired *t*-test, Welch’s test, one-way analysis of variance (ANOVA), Mann–Whitney *U* test, Kruskal–Wallis test, and Wilcoxon’s signed-rank test are often used [55,56,57]. When seeking to establish relationships between quantitative variables, the options are reduced to regressions and correlations [58,59].

It is essential to identify the type of variable to select the appropriate statistical test. The choice of test depends, among other factors, on the nature of the variable. It is crucial to note that statistical tests vary depending on the type of data; tests for numerical and qualitative variables are different. Therefore, starting by identifying the type of variable is fundamental.

### 5.2. Determine If the Statistical Test Involves Comparing between Groups or Intends to Find the Relationship with Another Variable

The second step in selecting the statistical test involves determining whether a comparison between groups will be conducted or if the aim is to establish a relationship between variables of the same type. This stage is crucial as it allows for the definition of the approach and the selection of the most appropriate statistical tests.

When comparing a variable between groups, the research objective is to determine if significant differences exist or if the variables show similar behavior among the groups of interest. For example, if there is interest in comparing glucose concentrations between a group of patients with diabetes mellitus and hypertension and another group composed solely of patients with diabetes mellitus. In this case, if a numerical variable is to be compared, common statistical tests include two-sample *t*-test, paired *t*-test, Welch’s test, and ANOVA and its non-parametric variants (Mann–Whitney U test, Kruskal–Wallis test, and Wilcoxon’s signed-rank) [55]. However, as will be seen later, the choice of the test will depend on the number of groups and the nature of the data.

When the objective is to determine the relationship between two variables, common statistical techniques such as linear regression, logistic regression, Pearson correlation, and Spearman correlation are used. However, the choice of the appropriate technique will again depend on whether the variables are continuous or categorical, as well as the nature of the expected relationship (linear or non-linear). For example, when analyzing the relationship between fasting glucose concentrations and uric acid concentrations, one of these techniques would be selected based on the nature of the data and the type of expected relationship [58,59].

It is fundamental to note that when establishing differences between groups, different statistical tests should be used from those employed to examine the relationship between two variables without conducting comparisons between groups.

### 5.3. Determine If Measurements Will Be Taken in Different Groups or If Repeated Measurements Will Be Taken in the Same Group over Time

Another crucial aspect in selecting the statistical test is understanding the nature of the groups. It is essential to determine whether measurements will be taken within the same group over time or if they will involve independent groups, as this can influence the type of appropriate test. In the former case, when the comparison groups are related or paired, it means that measurements were taken on the same sample or individuals at different time points. For example, in cohort studies, urine protein concentrations can be evaluated both at the beginning of the study and after a specified period. In this scenario, it may be of interest to determine whether urine protein concentrations have increased, decreased, or remained unchanged at the end of the study compared to the beginning. For this type of group, commonly used statistical tests include the paired *t*-test and Wilcoxon’s signed-rank test for numerical variables and the McNemar test for qualitative variables [51,56,60].

On the other hand, when the groups are not related (i.e., independent), several statistical tests are available. For quantitative variables, these include the two-sample *t*-test, Welch test, ANOVA, Mann–Whitney U test, and Kruskal–Wallis, whereas for qualitative variables, the Chi-Square Test or Fisher’s Exact Test can be used [55].

These tests are useful in observational studies, such as using the independent samples *t*-test, which could be applied to compare characteristics or variables of interest between different demographic groups. For example, we could analyze the average incomes between men and women in a specific population.

In case–control studies, the independent samples *t*-test could also be employed to identify significant differences, such as in age, between cases and controls. On the other hand, the Chi-Square Test could be useful for comparing the proportion of women between cases and controls.

### 5.4. Establish the Number of Groups Being Compared

The proper selection of the statistical test crucially depends on the number of groups in which the variable will be compared. This factor significantly influences the interpretation of the results and, ultimately, the conclusions of the research. Therefore, it is essential to determine whether the comparison will be made between two groups or more than two groups, as this will guide the choice of the most appropriate test.

For example, when comparing numerical variables between two groups, the most appropriate options include the two-sample *t*-test, paired *t*-test, Welch’s test, Mann–Whitney U test, and Wilcoxon signed-rank test. It is crucial to note that both the paired *t*-test and the Wilcoxon signed-rank test allow for analyzing changes over time and, therefore, comparing paired or dependent groups. On the other hand, when comparing the numerical variable of interest among more than two independent groups, the most suitable options are ANOVA and the Kruskal–Wallis test [55,56,57,60,61].

In the case of qualitative variables, the selection of the test is generally more straightforward, as it is not influenced by the number of groups. This means that whether comparing two groups or more than two, the statistical test employed will be the same. For comparisons between independent groups, common options include the Chi-Square Test and Fisher’s Exact Test, while for dependent groups, the McNemar Test is used [50,52,61].

### 5.5. Identify the Distribution of the Data

Many statistical tests require certain assumptions to be met. Although addressing all these assumptions is beyond the scope of this review, one of the critical assumptions is the normality of the data. It is important to note that this assumption only affects the selection of statistical tests for numerical variables. Data are considered to follow a normal distribution when they meet the criteria of symmetry, kurtosis, unimodality, and size [62,63,64].

There are several tests to assess the normality of the data, such as the Kolmogorov–Smirnov test, Shapiro–Wilk test, Anderson–Darling test, and Lilliefors test. However, it is advisable to avoid the isolated use of these tests. It is preferable to gain a deeper understanding of the data distribution and complement normality tests with graphs, as well as assess skewness and kurtosis before deciding whether the data follow a normal distribution or not [62,63,64].

For data that follow a normal distribution or approximate it, the use of statistical tests considered within the group of parametric tests is recommended. These include the two-sample *t*-test, paired *t*-test, Welch test, ANOVA, simple linear regression, and Pearson correlation. On the other hand, if the data do not meet the normality assumption, it is recommended to use non-parametric statistical tests, which do not rely on assumptions about the underlying data distribution. These tests include the Mann–Whitney *U* test, Kruskal–Wallis test, Wilcoxon’s signed-rank test, and Spearman correlation coefficient [62,63,64].

It has been reported that one of the main problems related to the statistical aspect of many articles is the use of inappropriate statistical tests according to the data distribution [50]. Therefore, it is of utmost importance to conduct a thorough evaluation of the data before proceeding with any statistical analysis in research. Additionally, having a complete understanding of the data being examined is essential.

### 5.6. Define the Type of Study

The identification of the type of epidemiological study and its relationship with statistical tests conditions the selection of an appropriate hypothesis test, significantly impacting the interpretation and conclusions of the results. In the context of the epidemiological studies reviewed in this research (cross-sectional, case–control, cohorts), incorrect selection of statistical tests can lead to misinterpretations, affecting both the internal and external validity of the results and, at times, limiting their scope [65].

Below are some recommendations to consider when selecting the most appropriate statistical analyses according to the type of study. In cross-sectional studies, it is common to use descriptive and bivariate analyses, such as Chi-Square Tests, *t*-tests, and correlations, to explore associations between variables. Although, in certain cases, the use of regression analysis is justified in a cross-sectional study, it is essential to bear in mind that the interpretation of these tests should never extend beyond the limits of the cross-sectional study and should not address aspects related to temporality. In other words, in a cross-sectional study, we can assess factors associated with a condition, but we cannot estimate risks since predictions about incidence cannot be made [17,66].

In case–control studies, the design allows for the estimation of odds Ratios as the main statistical test. It is crucial to ensure that odds Ratios are presented with confidence intervals, as these provide additional information about the variability and true magnitude of the outcome [67,68].

On the other hand, in cohort studies, thanks to their design, it is possible to estimate incidences and measures of risk, such as relative risk. The lack of estimation of these measures can significantly limit the interpretation and scope of a cohort study. For relative risk, it is also advisable to review and report confidence intervals, as these provide information about the magnitude and variability of the risk. Although the interpretation of relative risk and odds ratio can be complex, it is important not to confuse the interpretation of these measures. The odds ratio is interpreted in the context of probability, while relative risk is interpreted as a direct risk. For example, the odds ratio represents the probability that individuals with the event were exposed, while relative risk indicates the risk of developing the event because of exposure to a certain factor [67,69].

It is important to consider that the information described in this last section may be subject to exceptions and should always be complemented with the context of the research and other study characteristics such as sample size, variability, variable characteristics, limitations, and objectives.

## 6. Conclusions

Epidemiological studies are essential for public health as they enable us to understand the distribution of diseases, identify their causes and risk factors, and develop effective methods for their control and prevention. Among epidemiological studies, cross-sectional, case–control, and cohort studies are the most used due to their ability to provide valuable information in various contexts.

Despite the availability of guidelines for the design and reporting of analytical observational studies, there are still significant opportunities for improvement in the quality of these studies. In this work, it was identified that errors in the classification of methodological design and the selection of statistical tests are common. The most frequently confused designs are case–control studies with retrospective cohorts and cross-sectional studies with case–control studies. There is also confusion when comparing case–control studies, which are observational studies, with experimental studies such as clinical trials. Another common error is assigning temporality to cross-sectional studies as if they were longitudinal studies. Regarding the selection of statistical tests, the most common error is the use of incorrect tests for the type of data.

It is crucial that enhancing the quality of publications becomes a collaborative effort involving reviewers, authors, and publishers. Authors should strive to present high-quality work, while publishers should provide clearer and more precise guidelines to facilitate the evaluation process. This work stems from the identification of these opportunities for improvement in the publications of analytical observational studies. Based on this premise, a series of methodological and statistical considerations are presented that complement and enrich existing guidelines, aiming to raise the standard of quality of research in this field.

## Figures and Tables

**Table 1:** Investigations that analyzed the misclassification of analytical observational studies.

Author and Year of Publication	Main Results
LeBrun, D. G., 2020 [12]	A total of 339 articles were identified and classified as case–control studies from 75 orthopedic journals. It was found that 227 were misclassified. The designs most commonly confused with case–control studies were cross-sectional and cohort studies.
Esene, I. N., 2018 [13]	Of 224 articles initially classified as case–control studies and published in 31 neurosurgery journals, it was determined that 91 of them were not actually case–control studies. Most of the incorrectly labeled studies corresponded to retrospective cohorts.
Kicielinski, K., 2019 [14]	Out of 125 articles classified as case–control studies and published in neurosurgery journals, it was observed that 79 were incorrectly labeled, with cross-sectional studies being the most commonly confused.
Mayo, N. E., 2009 [15]	Out of 86 articles classified as case–control studies published in rehabilitation journals, it was discovered that 56 of them were actually cross-sectional studies, and 13 were intervention studies.
Grimes D. A., 2009 [16]	In four journals, it was identified that out of 124 articles classified as case–control studies, 30% were mislabeled. The majority of mislabeled studies were actually retrospective cohorts

**Table 2:** Publications reporting to have conducted a prospective cross-sectional or retrospective cross-sectional study. The data presented in the table were obtained from Scopus [24]. Publication years are shown on the vertical axis, and the reported classification is shown on the horizontal axis. The search filtered documents that reported the words “cross-sectional-prospective” or “cross-sectional-retrospective” in the title, abstract, or keywords.

Year	Prospective Cross-Sectional	Retrospective Cross-Sectional
2023	136	423
2022	147	382
2021	136	337
2020	125	239
2019	106	221
